# Mitochondrial Chronic Progressive External Ophthalmoplegia

**DOI:** 10.3390/brainsci14020135

**Published:** 2024-01-27

**Authors:** Ali Ali, Ali Esmaeil, Raed Behbehani

**Affiliations:** Neuro-Ophthalmology Unit, Ibn Sina Hospital, Al-Bahar Ophthalmology Center, Kuwait City 70035, Kuwait; ali.moh.behbahani@gmail.com (A.A.); ali.moh.esmaeil@gmail.com (A.E.)

**Keywords:** chronic progressive external ophthalmoplegia, CPEO, mitochondrial diseases

## Abstract

Background: Chronic progressive external ophthalmoplegia (CPEO) is a rare disorder that can be at the forefront of several mitochondrial diseases. This review overviews mitochondrial CPEO encephalomyopathies to enhance accurate recognition and diagnosis for proper management. Methods: This study is conducted based on publications and guidelines obtained by selective review in PubMed. Randomized, double-blind, placebo-controlled trials, Cochrane reviews, and literature meta-analyses were particularly sought. Discussion: CPEO is a common presentation of mitochondrial encephalomyopathies, which can result from alterations in mitochondrial or nuclear DNA. Genetic sequencing is the gold standard for diagnosing mitochondrial encephalomyopathies, preceded by non-invasive tests such as fibroblast growth factor-21 and growth differentiation factor-15. More invasive options include a muscle biopsy, which can be carried out after uncertain diagnostic testing. No definitive treatment option is available for mitochondrial diseases, and management is mainly focused on lifestyle risk modification and supplementation to reduce mitochondrial load and symptomatic relief, such as ptosis repair in the case of CPEO. Nevertheless, various clinical trials and endeavors are still at large for achieving beneficial therapeutic outcomes for mitochondrial encephalomyopathies. Key Messages: Understanding the varying presentations and genetic aspects of mitochondrial CPEO is crucial for accurate diagnosis and management.

## 1. Introduction

Chronic progressive external ophthalmoplegia (CPEO) is the most common manifestation of mitochondrial diseases and is characterized by bilateral symmetrical progressive ptosis and reduced ocular motility. CPEO can be isolated or accompanied by a clinical feature of systemic involvement of mitochondrial dysfunction (CPEO plus syndrome). The worldwide prevalence of CPEO is unknown; however, the incidence of CPEO is 1–2 per 100,000. In the United Kingdom’s cohort database, the estimated prevalence of CPEO recorded was 1 in 30,000 [1].

Von Graefe, in 1868, originally described CPEO, and later, in 1958, Kearns and Sayre first described their triad of CPEO, retinal degeneration, and heart block (Kearns–Sayre syndrome) [2]. Microscopical findings of pathological mitochondria in 1967 and ragged red fibers in 1972 [3,4], as well as increased venous pyruvate and lactate in 1976 [5], were significant in understanding the disease’s path. More recently, singular and multiple mitochondrial DNA (mtDNA) deletions were detected in 1988 and 1989 [6,7]. Finally, in 2000, the first nuclear DNA (nDNA) mutation was detected alongside multiple mtDNA deletions [8].

## 2. Pathophysiology, Genetics and Classification

**Figure 1:**
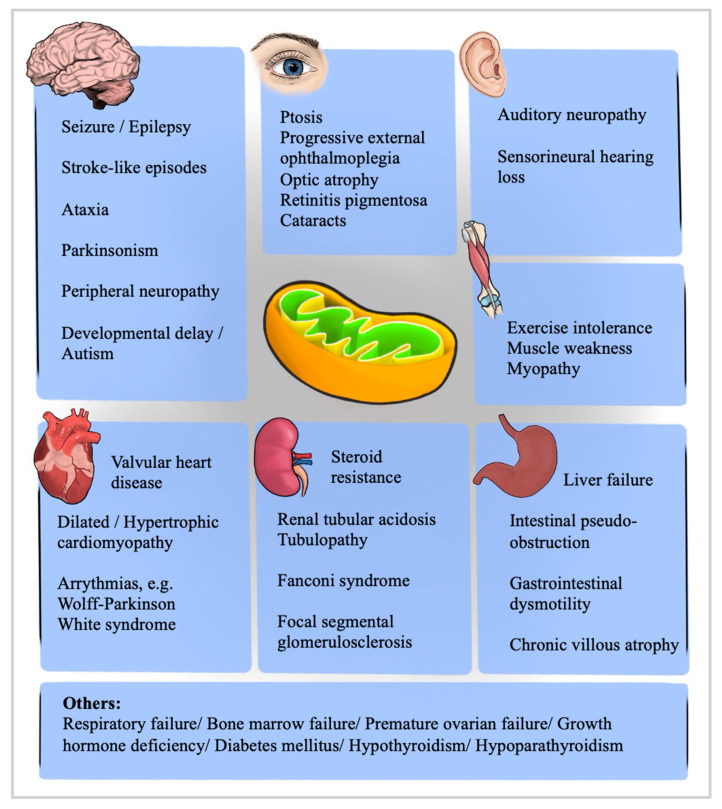
The diverse clinical outcomes of mitochondrial encephalomyopathies necessitate thorough screening of patients under suspicion.

Mitochondrial disorders generally affect tissues with high metabolic demand, such as the central and peripheral nervous systems, heart, adrenal glands, renal tubules, skeletal muscles, and the eye [9] (Figure 1). In CPEO, the ocular findings of ptosis and ophthalmoplegia occur due to the inability of the abnormal mitochondria to supply an adequate amount of ATP due to defective oxidative phosphorylation. The extraocular muscles are particularly susceptible due to their high mitochondrial volume and lower mutational threshold [10]. Their susceptibility is expressed in multiple mitochondrial disorders, highlighting the significance of examining other manifestations in patients with PEO.

Both mtDNA and nDNA can be affected in mitochondrial diseases. mtDNA has a unique trait of heteroplasmy since numerous copies are distributed in varying numbers to each oocyte before fertilization. This ensures a fluctuation of mutant mtDNA load in the characteristic maternal inheritance of mtDNA. Point mutations occurring in an asymptomatic mother have a chance of either being diluted to her child or transmitted with a higher load [11,12]. When mutated mtDNA loads exceed a certain threshold, symptoms occur, and, in this case, mitochondrial encephalopathy, lactic acidosis, and stroke-like episodes (MELAS) can be inherited. Large-scale deletions can occur during embryogenesis, giving rise to mtDNA deletion syndromes such as CPEO, Kearns–Sayre syndrome (KSS), and Pearson syndrome (PS). In the case of Leigh syndrome (LS), both mtDNA and nDNA can be affected, giving rise to a similar clinical phenotype with different genetic variants.

**Figure 2:**
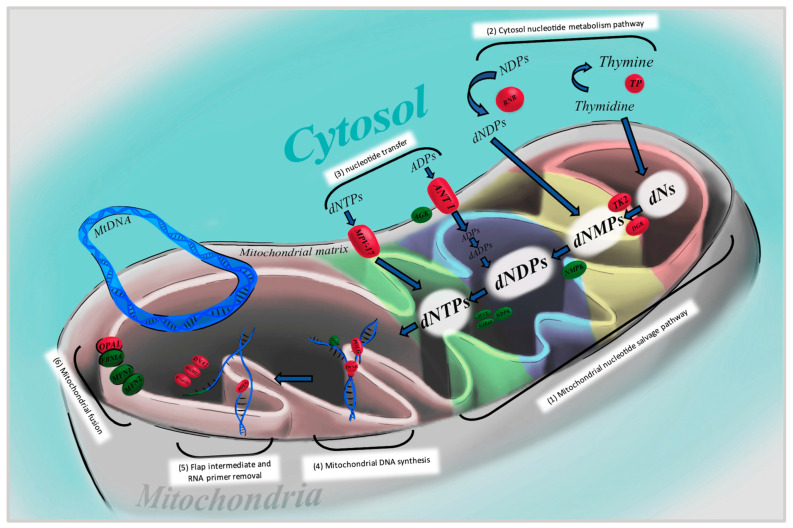
This diagram shows the proteins involved in the maintenance of mitochondrial DNA and the pathways involved. The mitochondrial nucleotide salvage pathway (1) is shown and is responsible for salvaging deoxyribonucleosides (dNs) and converting them into deoxyribonucleotide triphosphates (dNTPs) used in mtDNA replication. Along this pathway, Thymidine kinase 2 (TK2) and deoxyguanosine kinase (DGK) convert dNs into deoxyribonucleotide monophosphates (dNMPs) that later convert into deoxyribonucleoside diphosphates(dNDPs) by nucleotide monophosphate kinase (NMPK), then into dNTPs by nucleotide diphosphate kinase (NDPK). The cytosolic nucleotide metabolism pathway (2) includes thymidine phosphorylase (TP), which converts thymidine into thymine, and ribonucleotide reductase (RNR), which converts NDPs into dNDPs, supplying the nucleotide salvage pathway. RNR consists of two catalytic and two R2 or p53-induced small subunits. The nucleotide transport proteins (3) supply the nucleotide salvage pathway from the cytosol. MPV17 protein supplies dNTPs, while adenine nucleotide transporter (ANT1) supplies ADPs with the assistance of acylglycerol kinase (AGK), which are later converted to deoxyadenosine diphosphate (dADPs) feeding into the dNDPs. Mitochondrial DNA synthesis (4) requires the enzymes TWINKLE, a helicase, and the synthesis initiator, DNA polymerase gamma (POLG), which needs an RNA primer that is supplied by mitochondrial transcription factor A (TFAM). POLG consists of one catalytic subunit and two subunits encoded from POLG2. (5) The removal of RNA primers and flap intermediates is then achieved via ribonuclease H1 (RNase H1), DNA helicase/nuclease 2 (DNA2), and mitochondrial genome maintenance exonuclease 1 (MGME1). (6) Mitochondrial fusion is mediated by the proteins optic atrophy 1 (OPA1), F-box and leucine-rich repeat 4 (FBXL4), mitofusin 1 and 2 (MFN 1 and 2).

Mutations of nDNA can interfere with mitochondrial maintenance by affecting mtDNA synthesis, the mitochondrial nucleotide pool, and mitochondrial fusion. Nuclear genes responsible for synthesis include *POLG*, *TWNK*, *RNASEH1*, *MGME1*, and *DNA2*. The nucleotide pool is maintained through nucleotide metabolism with *TYMP* and *RRM2B*, the salvage pathway that includes *TK2* and *DGUOK*, and nucleotide import such as *ANT1* and *MPV17*. Finally, the *OPA1* gene aids in preventing the loss of mitochondrial components, contributing to mitochondrial fusion [13] (Figure 2).

In this review, we will review clinical entities of mitochondrial encephalomypoathies based on the corresponding genetics of each syndrome rather than symptomology because of the wide overlap of presentations and varying modes of inheritance accompanying each syndrome (Table A1).

## 3. Mitochondrial DNA Deletion/Depletion Disorders Causing CPEO

### 3.1. CPEO

**Figure 3:**
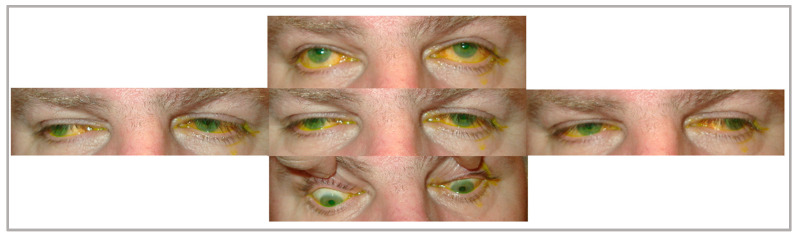
Patient A, diagnosed with mitochondrial encephalomyopathy, presents with chronic progressive external ophthalmoplegia with limited eye movements in all gazes and cerebellar signs (intention tremor in finger-to-nose test and tandem walking), in addition to areas of pigment hyperplasia on fundoscopy. The yellow discoloration shown in the image is from fluorescein eye staining.

In its isolated form, CPEO is typically a sporadic disorder characterized by progressive bilateral ptosis and ophthalmoparesis [9] (Figure 3). Ptosis examination yields poor levator palpebrae superioris (LPS) muscle function, where eyelid excursion is often less than 8–10 mm rather than the normal ≥12 mm. Slowed, incomplete, and omnidirectional saccades can be a subtle early clinical sign that is frequently missed. Later on, as the disease progresses, ophthalmoplegia becomes more evident. The often-symmetric nature of the disease means that patients do not have diplopia, and reports of manifest strabismus with diplopia in CPEO patients are rare [14,15]. Retinal examination could reveal pigmentary retinopathy that is typical in Kearns–Sayre syndrome, characterized as salt and pepper retinopathy, where clumps of retinal pigment epithelium (RPE) alternate with areas devoid of RPE [16]. However, these retinal changes rarely harbor field defects or a change in visual acuity.

Treatment of CPEO is focused on the correction of ptosis. It can start with eyelid crutches as a non-surgical solution, which usually is not preferred due to discomfort or intolerable aesthetics [17]. Surgery is the mainstay treatment and is dependent on LPS function. Resection of the levator tendon along the superior tarsus is available for mild LPS impairment, while in more severe cases, frontalis suspension procedures with facia lata or silicon are used [18,19]. When strabismus and diplopia occur, prismatic glasses are prescribed to correct small malalignments, and strabismus surgery can be performed to improve the patient’s quality of life [19].

### 3.2. Kearns–Sayre Syndrome

Kearns–Sayre syndrome is a syndrome of CPEO and pigmentary retinopathy, with onset before the age of 20 as well as one of the following features: a complete heart block, cerebellar ataxia, dementia, deafness, short stature, endocrine abnormalities, and cerebrospinal fluid (CSF) protein of more than 100 mg/dL. If the diagnostic criteria are not met, the patient is termed “CPEO plus” or “KSS-minus” [20].

When a patient presents with CPEO before the age of 20, they should be evaluated with mtDNA sequencing followed by regular ophthalmologic assessments and screening for systemic signs and symptoms. A muscle biopsy can be performed to look for the ragged red fibers. The fundoscopic examination reveals pigmentary retinopathy that should be distinguished from retinitis pigmentosa since they might share similar symptoms like mildly reduced night vision and visual acuity. Retinitis pigmentosa typically affects the peripheral or the mid-peripheral retina with a bone spicule pattern, whereas KSS affects the posterior retina with a salt and pepper pattern [21]. It is essential to perform an electrocardiogram on these patients to rule out a complete heart block. Endocrine abnormalities affecting the adrenals, parathyroid, and hypothalamus can present with diabetes mellitus, growth hormone deficiency, and short stature [22,23]. Orbicularis oculi muscle weakness can impair eyelid closure, and frontalis weakness can affect eyelid elevation. Dysphagia is a rare presentation of KSS and may result from upper esophageal sphincter dysfunction and reduced peristalsis in the pharynx and upper esophagus, as observed in a manometric study of a case report by Shaker et al. [24].

No definitive treatment option is available for KSS. Symptomatic treatment includes correction of CPEO, treating heart blocks with pacemakers with a long-term cardiology follow-up, correction of endocrine abnormalities, and cochlear implants in cases of hearing loss.

### 3.3. Pearson Syndrome

Pearson syndrome (PS), also known as Pearson marrow–pancreas syndrome, is a rare fatal multisystemic mitochondrial disease due to deletions in mtDNA, and it typically affects infants. Ophthalmologic manifestations include corneal endothelial dysfunction, ptosis, CPEO, and mild peripheral pigmentary retinopathy [25]. It is also characterized by refractory sideroblastic anemia, lactic acidosis, and exocrine pancreatic dysfunction. It can also present with vacuolization of hematopoietic precursors, pancytopenia, failure to thrive, diarrhea, hypospadias, cleft lip palate, diabetes mellitus, renal tubular dysfunction, hepatic failure, enteropathy, and rashes [26]. Cardiac manifestations, such as bundle branch blocks and supraventricular tachycardia, have been reported; however, cardiac involvement is not yet a part of the major criterion of the disease [27].

Usually, premature death at three years of age occurs due to infection from neutropenia or metabolic crisis. Thus, early diagnosis is essential in improving the poor prognosis for these patients. The diagnosis of Pearson syndrome is challenging due to the atypical presentation in infancy. It can be confirmed via mtDNA sequencing and observing multiple deletions of varying lengths [28]. Interestingly, these single large-scale mtDNA deletions can also be found in young patients with CPEO and KSS. They, therefore, form a continuous spectrum of diseases termed “mtDNA deletion syndromes”, supported by reports of a KSS-like phenotype in PS survivors [29].

Treatment for Pearson syndrome is supportive and may include blood transfusions, iron chelating therapy, pancreatic replacement therapy, and prompt detection and management of cardiac dysfunction. Bone marrow transplant has been tested and, unfortunately, yielded poor outcomes [26,30].

### 3.4. Leigh Syndrome

Leigh syndrome is a fatal, progressive neurodegenerative disease that typically manifests in infants and young children of 3 months to 2 years of age [31]. It can be caused by multiple mtDNA deletions as well as nDNA defects in more than 75 different monogenic causes, most commonly by the *SURF1* variant [32,33].

The clinical features of LS vary, with the most common characteristics, according to a meta-analysis by Chang et al., being developmental delay, hypotonia, respiratory dysfunction, epilepsy, reduced feeding, and weakness [34]. The ocular features of LS include nystagmus, ptosis, ophthalmoplegia, strabismus, pigmentary retinopathy, and optic atrophy [34,35]. Common cardiac abnormalities are hypertrophic or dilated cardiomyopathy and conduction defects such as Wolff–Parkinson–White syndrome [36,37].

Consensus on the clinical diagnosis is yet to be determined; however, LS is suspected through the hallmarks of the disease along with findings suggestive of brainstem dysfunction in addition to T2 weighted brain MRI lesions and accessory laboratory findings [34]. Brain MRI findings typically show bilateral symmetrical supra-tentorial (basal ganglia, thalamus, and sub-thalamus) and/or infra-tentorial (brainstem and dentate nuclei) lesions. A study by Ardissone et al. presented a predominating basal ganglia involvement of 90.2%. They also showed that both supra and infra-tentorial involvement is dominant in cases of both mtDNA (74%) and -nDNA (67%) variants, while isolated infra-tentorial variants are rare [38]. Extensive research is being conducted to find genetic correlations with MRI findings of LS. For example, a retrospective cohort found significant associations between the *SURF1* variant and inferior olivary nuclei lesions [39].

Abnormal laboratory findings may yield elevated blood, urine, and CSF lactate levels. Additional deficiencies may be observed in respiratory chain complexes through enzyme assays and pyruvate dehydrogenase complex [40]. However, these laboratory findings are not consistently present. Therefore, confirmatory tests with genetic assays are required for a definitive diagnosis and the identification of specific variants of LS [41].

### 3.5. MELAS

MELAS, or mitochondrial encephalopathy, lactic acidosis, and stroke-like episodes (SLEs) are associated with A to G RNA transfer mutation (Leu (UUR)) in the most commonly *m.3243A>G* mutation [42,43].

The clinical presentations vary widely, usually in childhood, with neurological symptoms that include SLEs, sensorineural hearing loss, and cognitive impairment associated with diffuse white matter injury. Less commonly, it can present with gastrointestinal manifestations that include gastric perforation, ischemic colitis, segmental ileal paralysis, pseudo-obstruction, or megacolon. Endocrine manifestations, such as diabetes mellitus, have also been reported in MELAS [44].

Ophthalmologic manifestations of MELAS include hemianopia and cortical blindness from SLEs, nystagmus, cataracts, CPEO, optic atrophy, salt and pepper pigmentary retinopathy, and macular degeneration [45].

The transient SLEs of the disease are characterized by nausea, vomiting, a migraine-like headache, encephalopathy, and focal seizures with or without neurological deficits. The exact pathogenic mechanism for these episodes is yet to be determined; however, three theories have been postulated. The first is insufficient energy due to mitochondrial dysfunction, supported by the increase in lactate peaks and decreased N-acetyl aspartate peaks of the occipital regions in brain magnetic resonance spectroscopy (MRS) [46]. The second is nitric oxide (NO) deficiency, which usually regulates oxygenation and blood flow. This hypothesis is supported by a reduction in NO metabolites during acute attacks and an increase in NO synthase inhibitors in the COX-negative fibers of MELAS patients [47]. The third theory is mitochondrial angiopathy, an accumulation of mitochondria in the smooth muscle cells and endothelial cells of small cerebral arteries leading to the narrowing of the lumen of blood vessels and reducing perfusion [48]. MRI findings of SLE exhibit stroke-like lesions (SLLs) that are usually differentiated from other pathologies by initially observing cortical and deep white matter lesions, in addition to occipital and parietal lobe lesions or lesions not confined to arterial territories. PWI/ASL can also show hyperperfused lesions, and MRS exhibits lactate peaks [49]. Another distinctive finding in neuroimaging was reported in some cases of MELAS as cerebellar lesions SLLs [49,50].

Since MELAS is associated with reduced levels of citrulline and arginine, which are NO precursors, and decreased NO that contributes to SLEs, supplement replacement with arginine was proposed. A systematic review by Argudo et al. concluded that the studies conducted showed promising results in managing SLEs [51]. Acute phase management consists of giving an intravenous dose of 500 mg/kg/day or 10 g/m^2^ in 24 h for 3–5 days. Whereas chronically, 150–300 mg/kg/day (maximum of 500 mg) is used instead [52]. A study conducted by Pek et al. using induced pluripotent stem cell-derived endothelial cells vouched for edaravone, a potent antioxidant, to be used for improving the vascular function in MELAS since it scavenges ROS and inhibits the inflammatory response in cerebrovascular diseases, which L-arginine and citrulline do not tackle [42]. For treating epilepsy, levetiracetam is considered to be the first-line anticonvulsant in mitochondrial encephalomyopathy due to the mitochondrial toxicity of other anticonvulsant agents [53].

## 4. Nuclear DNA Gene Mutations/Protein Dysfunction Causing CPEO

### 4.1. DNA Polymerase Subunit Gamma (POLG)

*POLG*-related disorders affect the nDNA that encodes mtDNA polymerase gamma. The first identified *POLG* mutation variant was inherited in an autosomal dominant manner; however, it was identified in recessive variants later in other families. CPEO was observed in both autosomal dominant and recessive carriers along with other neurodegenerative disorders associated with *POLG* that include myoclonic epilepsy myopathy sensory ataxia (MEMSA), childhood myocerebrohepatopathy spectrum (MCHS), Alpers syndrome, Alpers–Huttenlocher syndrome (AHS), and ataxia neuropathy spectrum (ANS) disorder [54].

The clinical presentation of *POLG*-related disorders can vary widely and include neurological features such as ataxia, axonal neuropathy, myoclonic epilepsy, and sensorineural hearing loss. Other features are PEO, cataracts, hypogonadism, liver dysfunction, and possible renal manifestations [55].

The diagnostic approach of *POLG*-related disorders proposed by Hikmat et al. is simplified and accounts for the age of onset and clinical picture with supportive and definitive investigations. EEG, MRI, muscle biopsy, and laboratory investigations are used as supportive investigations and ordered depending on the clinical presentation. *POLG* gene sequencing is the definitive investigation [55].

### 4.2. Twinkle mtDNA Helicase (TWNK)

The *TWNK* gene, also known as *C10orf2* or *PEO1*, is responsible for encoding TWINKLE, an mtDNA helicase, an enzyme that unwinds DNA temporarily for replication [56]. The dysfunction in TWINKLE is thought to pause or stall mtDNA replication and accumulate many mtDNA deletions over time [57]. Mutations in this gene are usually associated with adult-onset autosomal dominant PEO (adPEO). The clinical presentation can be isolated CPEO or with systemic muscle weakness, dysarthria, dysphagia, and cardiac or neurological involvement [58]. Treatment options for this disease are supportive and focused on alleviating symptoms.

### 4.3. Thymidine Phosphorylase

The *TYMP* gene encodes thymidine phosphorylase (TP), an enzyme that catalyzes thymidine and deoxyuridine into thymine and uridine, respectively. Defects in this gene cause the accumulation of thymidine and uracil in the blood, resulting in mitochondrial neurogastrointestinal encephalopathy (MNGIE). MNGIE is a rare multisystemic autosomal recessive disorder that typically starts before the second decade of life but can manifest up to the fifth decade [59,60]. It is characterized by CPEO, cachexia, severe gastrointestinal dysmotility, sensorineural hearing loss, peripheral neuropathy, and leukoencephalopathy [59]. Clinically, diagnosis can be supported by increased plasma levels of thymidine (>3 micromol/L) and deoxyuridine (>5 micromol/L) or a decrease in the buffy coat of TP activity to less than 8% of controls [61]. It is important to note that *TYMP* is not the only mutation attributed to a MNGIE-like phenotype, as *POLG1*, *RRM2B*, and *LIG3* mutations were reported with a somewhat similar clinical phenotype in the literature [62,63]. This can be of therapeutic value since *TYMP* mutations have ongoing treatment modalities to restore TP activity and target the toxic effects of thymidine and uracil, such as platelet infusion, continuous ambulatory peritoneal dialysis, enzyme replacement therapy, hematopoietic stem cell transplantation, and liver transplantation [62].

### 4.4. Ribonucleoside–Diphosphate Reductase Subunit M2 B (RRM2B)

Ribonucleotide–diphosphate reductase subunit M2 B is an enzyme encoded by *RRM2B*, which produces one of the two subunits of ribonucleotide reductase. Ribonucleotide reductase is induced by p53 to produce deoxyribonucleoside diphosphatase, a nucleotide precursor, for DNA repair and mtDNA synthesis in non-proliferating cells [64].

Defects in this gene can cause mtDNA maintenance defects, either from mtDNA depletion in *RRM2B* encephalomyopathic mitochondrial DNA maintenance defect (MDMD) and in *RRM2B* mimicking mitochondrial neurogastrointestinal encephalopathy (MNGIE) or multiple mtDNA deletions in *RRM2B* adPEO and *RRM2B* autosomal recessive PEO (arPEO) [65].

A common feature in these subtypes is ophthalmoplegia and ptosis; however, the age of onset and other clinical features may differ. For example, *RRM2B* adPEO usually accompanies bulbar dysfunction, hearing loss, and gastrointestinal motility [64]. On the other hand, arPEO is a childhood-onset disease that is more severe with associated retinopathy, myopathy, and mood disorders [64,66]. *RRM2B* encephalomyopathic MDMD is an infantile-onset severe multisystem disease that usually presents with hypotonia, poor feeding, and failure to thrive. In addition, other manifestations include respiratory failure, renal tubular necrosis, and sensorineural hearing loss [67]. Finally, MNGIE-like *RRM2B* is a rare phenotype that can occur with cachexia, gastrointestinal dysmotility, and peripheral neuropathy [68].

### 4.5. Optic Atrophy 1 (OPA-1)

The *OPA-1* (optic atrophy 1) gene is a membrane-remodeling protein that regulates mitochondrial dynamics with both energetics and mitochondrial morphology [69]. It was discovered in 2000, along with its association with dominant optic atrophy (DOA). Since then, a broad spectrum of clinical features have been reported in DOA plus syndromes that include Behr syndrome, syndromic parkinsonism, dementia, CPEO, and other neuromuscular features [70,71,72]. The clinical features characterizing DOA are bilateral progressive visual loss involving color vision and the central or paracentral visual field with varying severity. Funduscopic examination exhibits optic pallor or atrophy related to retinal ganglionic cell (RGC) layer death. Additional diagnostic modalities for DOA can involve optical coherence tomography, which shows nonspecific retinal nerve fiber layer thinning and abnormal visual evoked potentials due to RGC dysfunction [73]. In a cohort by Romagnoli et al. for using Idebenone as a therapeutic option for *OPA1*-DOA, patients who underwent the therapy benefited in terms of visual recovery four times more than those who did not. This shows promising results that have yet to be confirmed by future studies [74].

### 4.6. Thymidine Kinase 2 (TK2)

The Thymidine kinase 2 (*TK2*) gene encodes for an enzyme integral for mtDNA replication and maintenance since it phosphorylates deoxythymidine (dT) and deoxycytidine (dC) into deoxynucleotide triphosphates in the deoxypyrimidine salvage pathway [75]. The clinical picture of TK2 deficiency (TK2d) varies with the age of onset and is categorized into early-onset (≤1 year), childhood-onset (>1 to ≤12 years), and late-onset (>12 years) TK2d.

Early-onset TK2d is usually a severe myopathic form that is fatal within a year, with early symptoms preceding muscle weakness that include esophageal reflux, vomiting, intestinal dysmotility, and failure to thrive. These patients can also exhibit neurological and extra-skeletal manifestations, which include seizures, cognitive impairment, bilateral optic atrophy, multiple fractures, rigid spine, nephropathy, and cardiomyopathy.

The childhood-onset form of the disease typically has an intermediate to a rapidly progressive phenotype of proximal myopathy with Gowers signs and a dropped head. Some cases can also show CPEO, facial diplegia, dysphagia, and restrictive lung disease, aiding the diagnosis. Extra-skeletal findings may include multiple fractures, cognitive decline, encephalopathy, hearing loss, renal tubulopathy, and arrhythmias.

Patients with late-onset TK2d have the characteristic progressive proximal muscle wasting, with the addition of axial neck flexors and facial weakness. This form usually accompanies CPEO, bulbar weakness, and early respiratory muscle involvement requiring non-invasive ventilation. In some cases, peripheral neuropathy and hearing loss can also occur [76].

A recent cohort by Domínguez-González et al. demonstrated a characteristic lower limb muscle MRI pattern that can differentiate the condition from other myopathies with similar clinical features [77].

Therapy using the active substrates dT and dC in TK2d patients has been reported to improve muscle weakness and ambulation, as well as discontinuing mechanical ventilation and gastrostomy in affected children. However, late-onset cases showed minimal benefits, and further studies are needed to establish a clear benefit [78].

### 4.7. Deoxyguanosine Kinase

Deoxyguanosine kinase (DGK) phosphorylates purine deoxyribonucleosides and contributes to the deoxyribonucleoside salvage pathway in the mitochondrial matrix [79]. Two forms of *DGUOK* gene deficiency have been described in the literature, with neonatal multisystem disorder being the most common [80]. It is characterized by hepatic and neurological manifestations, including developmental delay, hypotonia, nystagmus, jaundice, cholestasis, and hepatomegaly [81]. The second less severe form is an isolated childhood hepatic disorder. Long-term follow-up of varying cases with this phenotype showed renal involvement, myopathy, and parkinsonism with CPEO, rigidity, and bradykinesia [82,83,84]. The most common cause of mortality in both forms is progressive hepatic disease, and a decision on whether a transplant is needed should be as per hepatologist since the topic is under debate [81,85].

### 4.8. Ribonuclease H1 (RNase H1)

RNase H1, or ribonuclease H1, is an enzyme encoded by the gene *RNASEH1*, located in chromosome *17p11.2*. This enzyme contributes to mitochondrial dynamics through primer maturation, removal, synthesis of replication primer, and pre-RNA processing in mtDNA replication [86,87]. In a cohort conducted by Bugiardini E. et al., patients harboring *RNASEH1* mutations had characteristic features of CPEO, cerebellar ataxia, and dysphagia, with CPEO being a universal feature in all cases. In contrast, ataxia and dysphagia were concomitantly present in approximately 50% of cases. Other less frequent symptoms were proximal muscle weakness, peripheral neuropathy, and pyramidal signs. This study also concluded that in the presence of *POLG*-negative ataxia neuropathy spectrum, all patients should be considered for genetic analysis for *RNASEH1* mutations since it is the fourth most common cause of adult mendelian PEO with multiple mtDNA deletions in their cohort, following *POLG*, *TWNK* and *RRM2B* [88]. Manini et al. reported similar findings in their case report and compiled data from several reports of patients with the *RNASEH1* mutation and noted that some of these frequent findings have been observed in other mitochondrial diseases, such as dysarthria in adults with *POLG* and *TK2* mutations, and cerebellar signs in late-onset *RRM2B* mutations [87].

### 4.9. Mitochondrial Genome Maintenance Exonuclease 1 (MGME1)

**Figure 4:**
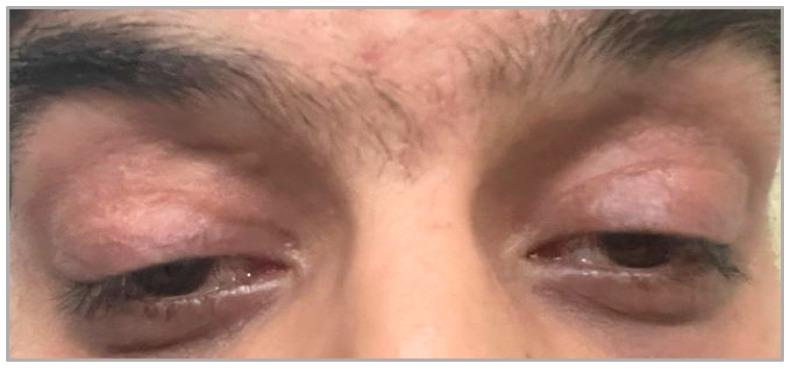
Patient B is a 27-year-old male with a recent diagnosis of a homozygous pathogenic variant of MGME1, presenting with chronic progressive external ophthalmoplegia (limitation with horizontal and vertical gazes), refractory errors, pigmentary retinopathy, exercise intolerance, myopathy, fatigue, attention-deficit/hyperactivity disorder, and right bundle branch block. He underwent ptosis repair at the ages of 15 and 17, but ptosis recurred over time. He has a long family history of consanguinity and a similar clinical phenotype presented in his cousin and past uncles.

Mitochondrial genome maintenance exonuclease 1 (*MGME1*), or Ddk1, is an exclusive mitochondrial DNase responsible for mtDNA maintenance by preferentially cutting single-stranded DNA (ssDNA) flaps and enabling the ligation of new DNA strands [89]. *MGME1* affects the turnover of 7S DNA and causes its accumulation when depleted or causes 7S DNA attrition when overexpressed [90]. 7S DNA is a ssDNA arising from the non-coding region and is postulated to contribute to the mtDNA displacement loop (D-loop) as an intermediate of premature termination of mtDNA replication [91]. Patients with this mutation have shown an increase in 7S DNA and a multisystemic phenotype of PEO, muscle wasting, emaciation, and respiratory failure [92]. A similar phenotype was reported recently with skeletal malformations, atrioventricular block, and cerebellar atrophy in magnetic resonance imaging [93] (Figure 4).

### 4.10. Adenine Nucleotide Translocator 1 (ANT1)

*ANT1*-related PEO is associated with adPEO and affects the adenine nucleotide translocase type 1 (*ANT1*) gene, which encodes the translocator responsible for ADP to ATP exchange in the inner mitochondrial membrane and regulates the mitochondrial permeability transition pore that initiates apoptosis [94]. The complete loss of this gene causes the characteristic clinical phenotype of cardiomyopathy and myopathy [95]. On the other hand, the overexpression of this gene results in cardioprotective features [96]. Other clinical features of this disorder include exercise intolerance, muscle weakness, ptosis, and lactic acidosis [97]. All of the symptoms mentioned assist in suspecting the diagnosis, which can later be confirmed with genetic testing. There is no consensus on the treatment of this disorder. Standard heart failure treatment has been used to manage some cases to tackle cardiomyopathy; however, the results did not show any benefit in halting disease progression [98]. Recent studies suggest that reducing protein leak can be an effective treatment option for aged cardiomyocytes, which may assist in *ANT1*-related symptoms [99]. Another showed improvement in exercise intolerance using nicotinamide riboside in *ANT1*-deficient mice [100].

### 4.11. Mitochondrial Inner Membrane Protein MPV17

*MPV17* is an inner mitochondrial non-selective channel that is thought to play a role in mitochondrial maintenance by preventing the formation of reactive oxygen species [101,102]. The clinical picture of this mutation is similar to *DGUOK* with an early-onset hepatocerebral phenotype with hypoglycemia, metabolic acidosis, gastrointestinal findings of poor feeding, failure to thrive, and dysmotility. Rare cases of this mutation with a late-onset neuromyopathic phenotype have also been reported [103]. Brain MRI findings may harbor abnormalities in lower brainstem reticular formation, reticulospinal tracts at the cervicocranial junction, and cerebral leukoencephalopathy [102,104].

## 5. A Diagnostic Approach to Mitochondrial Encephalomyopathies

There is no consensus on a specific algorithm for diagnosing mitochondrial encaphalomyopathies, and a general approach of clinical suspicion was followed by clinical and biochemical findings, which were then forwarded to targeted or exploratory sequencing. Clinical suspicion requires knowledge of inheritance, varying phenotypical findings, and syndromes along the myopathic, encephalomyopathic, hepatocerebral, and neurogastrointestinal forms, among others. When clinical suspicion arises, an array of clinical and biochemical tests can be carried out to narrow down differential diagnoses and focus on targeted sequencing (Table 1).

Serum biomarkers of growth differentiation factor 15 (GDF-15) and fibroblast growth factor 21 (FGF-21) were proposed to aid in decision making as they increase in metabolic diseases with oxidative stress and inflammation; however, they cannot be used as a diagnostic tool for mitochondrial disorders as they increase in a variety of other non-mitochondrial diseases [105]. Additionally, GDF-15 was noted to be the most useful first-line test for mitochondrial respiratory chain deficiency, with a superior diagnostic sensitivity and odds ratio compared to FGF-21 [106,107]. Other biomarkers are plasma lactate for metabolic crisis, creatine kinase for myopathy, an endocrinological panel for diabetes, thyroid and parathyroid screening, and a urine dipstick with a renal function test. Neuroimaging, electroencephalography, and nerve conduction studies are used to screen and assess neuropathies. Cardiomyopathies are less frequent but require screening nonetheless with echocardiography, in addition to an electrocardiogram for arrhythmias. Muscle biopsies are less commonly performed now due to genetic testing being the reliable gold standard of diagnosis. They are used in some cases of unclear genetic testing or phenotype with the typical findings of ragged red fibers with trichome histological staining, which represent excess mitochondrial proliferation, and cytochrome c oxidase (COX) negative fibers in COX and succinate dehydrogenase stains [108].

**Figure 5:**
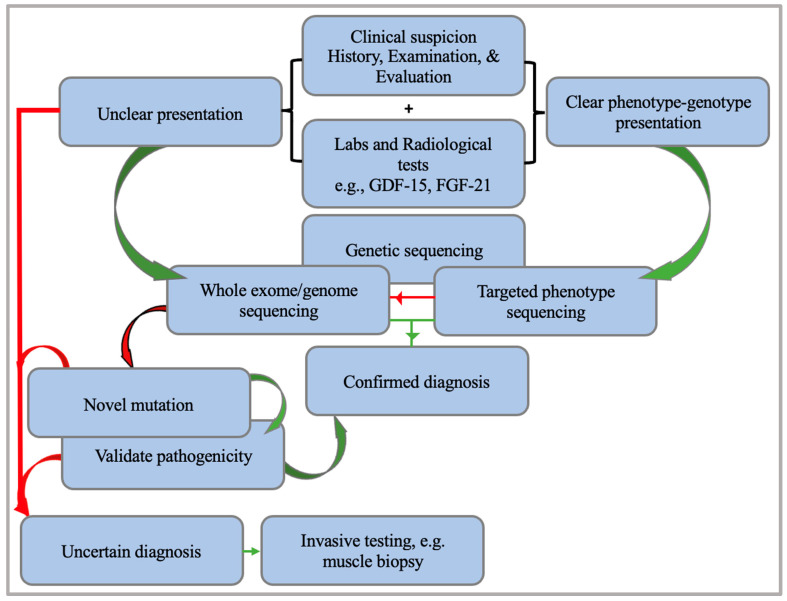
A summary of the proposed diagnostic pathway by Watson et al., where invasive testing is preceded by genetic sequencing, the gold standard of diagnosis. The green line indicates a yes, while the red line indicates no.

Next-generation sequencing (NGS) has been a significant development in diagnosing mitochondrial diseases, being faster, more accurate, and cost-effective. Watson et al. proposed a genetics-first approach towards confirming diagnoses using it along with ancillary non-invasive testing before moving to more invasive tests in case of unclear diagnoses. Their approach depends on identifying and running existing phenotype–genotype correlations through a targeted sequencing panel, followed by exploratory whole-exome (WES) or whole-genome sequencing (WGS) if no correlation was noted. This method also identifies novel mutations and confirms their pathogenicity; if not, a review or more invasive tests such as a muscle biopsy can be performed [109] (Figure 5).

## 6. Treatment of Mitochondrial Diseases

Patients with mitochondrial diseases are generally treated with supportive and symptomatic multi-disciplinary therapy. Regular aerobic exercise is recommended and thought to reduce fatigue and improve the quantity of muscle mitochondria and quality of life [110,111]. A ketogenic diet, which is high in fat and moderate in protein as well as low in carbohydrates, is an option for epileptics; however, it is contraindicated in patients with mtDNA deletion-related myopathy, so proper consultation with a nutritionist is recommended [112]. The patient should be counseled about avoiding toxic mitochondrial medications such as metformin, propofol, valproic acid, aminoglycosides, linezolid, and nucleoside analog treatments [113,114]. To counteract the impairment of mitochondrial function in these patients, a common treatment strategy employing a “mitochondrial cocktail” of vitamins, supplements, and antioxidants is used. These include L-carnitine, coenzyme Q10, riboflavin, thiamine, vitamin C, and E. [115]. Other pharmaceutical options used are Idebenone for *OPA1*, L-arginine and nicotinamide for MELAS, and active dT and dC substrates in TK2d (Table A2).

Options for future treatment by genetic therapy using mitochondrial genome manipulation in somatic tissues or replacement in the germline are still in the phase of clinical trials or animal models. Restriction endonucleases, transcription activator-like effectors, transcription activator-like effector nucleases, zinc finger nucleases, and clustered regularly interspaced short palindromic repeats all follow the concept of manipulating mtDNA through locating or targeting the mutation and then proceeding to eliminate or cleave the mutation. Replacement therapies that aim to replace mutated mtDNA with wild-type mtDNA include pronuclear and oocyte spindle transfer. They have been restricted in many nations due to debates and uncertainties about their outcomes [116].

## 7. Conclusions

In conclusion, understanding the pathophysiology of mitochondrial disorders has significantly advanced over the years, with the identification of mitochondrial and nuclear DNA mutations and their impact on different tissues and organs. This knowledge has led to improved classification and diagnosis of these disorders and to the knowledge that CPEO is one of their most common manifestations. Unfortunately, definitive phenotype–genotype correlations are still far out of reach, and physicians should familiarize themselves with these, maintain high clinical suspicion to diagnose them, and stop focusing on a solitary finding. Since no definitive treatment option is available, medical therapy focuses on alleviating symptoms and relieving defective mitochondrial expression. Genetic therapy is the future for treating these conditions, and this is an area where ongoing research is directed.

## Figures and Tables

**Table 1:** This table shows the differential diagnosis of chronic progressive external ophthalmoplegia, which should be excluded to avoid misdiagnosis.

Differential Diagnosis of Chronic Progressive External Ophthalmoplegia			
Myopathic	Neuropathic	Neuromuscular Junction	Other
Orbital myositis	Multiple sclerosis	Myasthenia gravis	Botulism
Graves’ disease	Miller Fisher syndrome	Congenital myasthenic syndrome	Medications: Statins
Myotonic dystrophy types 1 and 2	A-beta lipoproteinemia	Lambert–Eaton myasthenic syndrome (LEMS)	
	Tolosa-Hunt syndrome		
	WEBINO syndrome		
	CAPOS syndrome		
	CANOMAD syndrome		
Congenital myopathies	Supranuclear ophthalmoplegia: Hereditary ataxias HSP SCA 1, 2, 3, 7, 9, 11, 28 Congenital cranial dysinnervation disorders: CFEOM, Moebius syndrome, Duane syndrome		
OPMD			
OPDM			
LGMD with ophthalmoplegia			

Abbreviations: OPMD: Oculopharyngeal muscular dystrophy, OPDM: Oculopharyngodistal myopathy, LGMD: Limb-girdle muscular dystrophy, WEBINO: Wall-eyed bilateral internuclear ophthalmoplegia, CAPOS: Cerebellar ataxia, areflexia, pes cavus, optic atrophy, and sensorineural hearing loss, CANOMAD: Chronic ataxic neuropathy with ophthalmoplegia, IgM paraprotein, cold agglutinins, and disialosyl antibodies, HSP: Hereditary spastic paraplegia, SCA: Spinocerebellar ataxia, CFEOM: congenital fibrosis of the extraocular muscles.

## Appendix A

**Table A1:** The clinical features of mitochondrial encephalomyopathies.

Mitochondrial DNA Disorder	Acronym	Possible Clinical Features	Reference
Chronic progressive external ophthalmoplegia	CPEO	Progressive bilateral ptosis, ophthalmoplegia, diplopia	[117]
		CPEO “plus”: muscle weakness, exercise intolerance, short stature, pharyngeal muscle weakness, cognitive impairment, decreased vision, cardiac conduction block	
Kearns–Sayre syndrome	KSS	Criterion: CPEO and pigmentary retinopathy (onset < 20 years), cerebrospinal fluid protein >1 g/L. Plus one of the following: Cerebellar ataxia, myopathy, dysphagia, sensorineural hearing loss, heart block, diabetes mellitus, endocrine dysfunction	[118]
Pearson syndrome	PS	Sideroblastic anemia, pancytopenia, exocrine pancreatic failure, renal tubular defects	[119]
Mitochondrial encephalopathy, lactic acidosis, and stroke-like episodes	MELAS	Criterion: Stroke-like episodes (SLEs) (age < 40 years), seizures and/or dementia, signs of myopathy, e.g., ragged red fibers and/or lactic acidosis. Pigmentary retinopathy, diabetes mellitus, cardiomyopathy, bilateral sensorineural deafness	[120,121,122]
Leigh syndrome *	LS	Developmental delay, hypotonia, respiratory dysfunction, epilepsy, reduced feeding, weakness. Cardiomyopathy, cardiac conduction defects. Ptosis, ophthalmoplegia, nystagmus, strabismus, pigmentary retinopathy, optic atrophy	[34,35,36,37]
Maternally Inherited Leigh Syndrome	MILS	Intractable epileptic seizures, chorea, hyporeflexia, psychomotor retardation, hypoacusis, dystonia, hypotonia, hypertrophic cardiomyopathy, hepatopathy, lactic acidosis	[123]
Neuropathy, Ataxia, Retinitis Pigmentosa	NARP	Axonal neuropathy, cerebellar ataxia, proximal muscle weakness, retinitis pigmentosa. Epilepsy, cerebellar or cerebral atrophy, dementia, hypoacusis, optic atrophy, sleep apnea syndrome, renal impairment, diabetes mellitus	[124]
Leber’s hereditary optic neuropathy	LHON	Bilateral vision loss, central/centro cecal scotoma, mild disc pseudo edema, dyschromatopsia, peripheral neuropathy, clonus, dystonia, postural tremor, myopathy, Wolff Parkinson White syndrome	[125]
Maternally Inherited Diabetes and Deafness	MIDD	Diabetes mellitus, progressive sensorineural deafness, thin and short stature, macular dystrophy, proliferative retinopathy, myopathy, left ventricular hypertrophy, Wolff Parkinson White syndrome, atrial fibrillation, constipation, diarrhea, intestinal pseudo-obstruction	[126]
Myoclonic Epilepsy and Ragged Red Fibers	MERRF	Myoclonus, epilepsy, ataxia, migraine, dementia, SLEs, myopathy, myalgia, dysphagia, dysmotility, polyneuropathy, hearing loss, optic atrophy, ptosis, ophthalmoparesis, cardiomyopathy, arrhythmias, lipomatosis	[127]
Sensory ataxic neuropathy with dysarthria and opthalmoparesis *	SANDO	Ataxia, ptosis, dysarthria, sensory neuropathy, dysphagia, myalgia, seizures, diabetes mellitus	[128,129]
**Nuclear DNA gene**	**Protein**	**Possible clinical manifestations**	**Reference**
POLG	DNA polymerase subunit gamma	Seizures, ataxia, SLEs, peripheral neuropathy, migraine-like headache, hypotonia, liver involvement, anemia, ptosis, PEO	[55]
TWNK	Twinkle	Ataxia, neuropathy, myopathy, epileptic encephalopathy, CPEO, cataracts, mild cardiac abnormalities, parkinsonism	[58]
TYMP	Thymidine phosphorylase	Mitochondrial neurogastrointestinal encephalopathy (MNGIE): CPEO, cachexia, severe gastrointestinal dysmotility, sensorineural hearing loss, peripheral neuropathy, leukoencephalopathy	[59]
RRM2B	P-53 subunit of ribonucleotide reductase	Autosomal dominant PEO: Bulbar dysfunction, hearing loss, gastrointestinal dysmotility	[64]
		Autosomal recessive PEO: Retinopathy, myopathy, mood disorders	[66]
		Encephalomyopathy phenotype: Hypotonia, failure to thrive, sensorineural hearing loss, retinopathy, renal tubular necrosis, respiratory failure	[67]
		MNGIE-like phenotype: cachexia, gastrointestinal dysmotility, peripheral neuropathy	[67]
RNASEH1	Ribonuclease H1	CPEO, cerebellar ataxia, dysphagia Proximal muscle weakness, peripheral neuropathy, pyramidal signs	[87,88]
TK2	Thymidine kinase 2	Early-onset (≤1 year): Esophageal reflux, vomiting, intestinal dysmotility, failure to thrive, severe myopathy, seizures, cognitive impairment, rigid spine, multiple fractures, nephropathy, cardiomyopathy, bilateral optic atrophy Childhood-onset (>1 to ≤12 years): Gowers sign, dropped head, CPEO, facial diplegia, dysphagia, restrictive lung disease, encephalopathy, hearing loss, cognitive decline, multiple fractures, arrhythmias, renal tubulopathy Late-onset (>12 years): Proximal muscle wasting, facial and axial neck flexor muscle weakness, CPEO, bulbar and diaphragmatic weakness	[76]
DGOUK	Deoxyguanosine kinase	Psychomotor delay, hypotonia, nystagmus, Optic disc dysplasia, renal involvement, jaundice, cholestasis, hepatomegaly, progressive hepatic disease	[82,130]
		myopathy, parkinsonism, CPEO, rigidity, bradykinesia, progressive hepatic disease	[84]
OPA1	GTPase mitochondrial fusion	Progressive bilateral optic neuropathy, optic atrophy. Dominant optic atrophy-plus: CPEO, myopathy; ataxia; peripheral neuropathy sensorineural hearing loss	[70]
MGME1	Mitochondrial genome maintenance exonuclease 1	PEO, muscle wasting, emaciation, respiratory failure, skeletal malformations, atrioventricular block, cerebellar atrophy	[92,93]
ANT1	Adenine nucleotide translocator 1	Exercise intolerance, muscle weakness, ptosis, cardiomyopathy	[97]
MPV17	MPV17 protein	Early-onset hepatocerebral phenotype: hypoglycemia, metabolic acidosis, failure to thrive, liver failure, dysmotility, corneal scarring	[131]
		neuromyopathic phenotype	[132]
DNA2	DNA replication ATP-dependent	CPEO, limb-gridle weakness, Gowers sign, progressive muscle weakness, myalgia, and dyspnea	[133]
POLG2	DNA polymerase subunit gamma 2	Cerebellar ataxia, CPEO, neuropathy, seizures, parkinsonism, and exercise intolerance	[134]
AFG3L2	AFG3-like protein 2	Spinocerebellar ataxia type28, CPEO, optic atrophy, nystagmus, parkinsonism	[135,136,137]
SPG7	Paraplegin	Spastic paraplegia 7, Proximal myopathy, CPEO, optic atrophy, dysphagia, spasticity, ataxia, cerebellar atrophy	[138]
TOP3A	DNA topoisomerase 3 alpha	Cerebellar ataxia, neuropathy, sensorineural hearing loss, CPEO, myopathy, cardiac conduction defects	[139,140]
LIG3	DNA ligase 3	MNGIE and MELAS-like phenotype, headache, neurogenic bladder, cataracts, macular degeneration	[63]
RRM1	Ribonucleotide reductase catalytic subunit M1	Proximal myopathy, dysphagia, ptosis, ophthalmoparesis, peripheral neuropathy, nausea, vomiting, cachexia, intestinal dysmotility	[141]
C1QBP	Compliment C1q binding protein	Exercise intolerance, muscle weakness, CPEO, left ventricular hypertrophy	[142,143]
GMPR	Guanosine monophosphate reductase	CPEO	[144]

Ophthalmological features are underlined. * Can be associated with nuclear mutations.

**Table A2:** Proposed therapeutic options for mitochondrial encephalomyopathies.

Treatment	Mechanism of Action	Conditions	Results	Clinical Trial/Reference
Mitochondrial cocktail				
Coenzyme Q10 (ubiquinone)	Antioxidant/ electron flow restoration in the respiratory chain complex	Mitochondrial diseases	Phase 3 clinical trial (completed) No statistical difference in measured outcomes.	NCT00432744
		Primary and Secondary Coenzyme Q10 deficiency	Variable outcomes.	[145]
L-carnitine	Fatty acid metabolism, detoxification, cell membrane stabilization/control of ketogenesis/gluconeogenesis/Improves metabolic flexibility	Mitochondrial myopathy/CPEO	Improved exercise tolerance and aerobic capacity.	[146]
		Carnitine deficiency-related mitochondrial dysfunction	Maintenance of metabolic flexibility.	[147]
Riboflavin (Vitamin B2)	Electron carrier component	Primary and secondary flavoenzyme defects with mitochondrial dysfunction, e.g., complex 1 and 2 deficiencies	Dramatic improvements in supplementation for riboflavin-associated deficiencies.	[148]
Thiamine (Vitamin B1)	Coenzyme in the maintenance of carbohydrate metabolism	Genetic dysfunction of thiamine metabolism and transport.	Supplementation improves symptoms, clinical outcomes, and survival.	[149]
		Thiamine-deficient leigh syndrome	Significant reduction in morbidity and mortality.	[150]
Alpha lipoic acid (ALA)	Antioxidant	Age-associated cognitive and mitochondrial dysfunction	Improves age-associated memory loss, mitochondrial function, and structure.	[151]
		ALA and L-acetyl carnitine for supranuclear palsy	Phase 2 clinical trial (completed). Results posted and updated in 2017.	NCT01537549
		Parkinson’s disease	Slows cognitive decline effectively.	[152]
			Improved mitochondrial function and autophagy.	[153]
Folinic acid	Increases levels of 5MTHF levels in the brain, Folate is believed to play a role in mitochondrial nucleotide biosynthesis, and mtDNA replication	Cerebral folate deficiency in KSS	Reversal of leukoencephalopathy and normalization of 5MTHF CSF levels.	[154]
			Early, high dose treatment seems advisable in KSS.	[155]
		Cerebral folate deficiency and mitochondrial complex 1 deficiency	Improvement in hypotonia, ataxia, and cerebral hypomyelination.	[156]
Mito Q	Antioxidant targeting mitochondria	Mitochondrial diseases, e.g., Friedreich ataxia	Targeted antioxidants are more effective than non-targeting ones.	[157]
			Benefits including protection from ischemia/ reperfusion injury and endothelial damage.	[158]
Therapy tackling mitochondrial disease outcomes				
Idebenone (RAXONE)	Electron carrier, antioxidant	LHON	(RODOS study) No statistically significant difference in recovery of visual acuity except in discordant visual acuity Secondary endpoints were all statistically significant.	NCT00747487
			RODOS OFU study Beneficial effects persisted for a median of 30 months post discontinuation.	NCT01421381
			Phase 4 CT LOROS study Long term treatment results in prolonged clinical benefit in the subacute phase.	NCT02774005
		MELAS	Phase 2 clinical trial Non statistically significant difference in primary outcomes.	NCT00887562
Vatiquinone (EPI-743)	Inhibitor of 15-lipooxygenase, a regulator of oxidative stress pathways Augmentation of glutathione synthesis	Friedreich’s ataxia	Phase 2/3 clinical trial Non statistical difference in primary endpoints. However, there was a statistically significant impact in fatigue scores and disease progression of upright stability and bulbar subscales in a 72-week period.	NCT04577352
		Parkinson’s disease	Phase 2 clinical trial Statistically significant in retinal function and unified Parkinson’s disease rating scale. Decrease in oxidative stress markers in the basal ganglia with MRS.	NCT01923584
		Leigh syndrome	Phase 2 CT Fewer adverse events and hospitalizations in treatment group compared to placebo.	NCT02352896
Nicotinamide Riboside	Increasing intracellular levels of NAD, the crucial cofactor for mitochondrial energy production	Obesity and the related metabolic complications	Better systemic NAD metabolism, composition of gut microbiota, myoblast differentiation and mitochondrial number in the muscles.	[159]
		Healthy subjects	Increased NAD pools without apparent side effects.	[160]
		Parkinson’s disease	NADPARK study Phase 1 clinical trial Increased intracellular levels of NAD, lysosomal and proteasomal function of genes related to the mitochondria of blood and muscles. Decreased serum and CSF inflammatory cytokines.	NCT03816020
		ANT1-deficient mice	Increased exercise capacity and mitochondrial respiration.	[100]
L-arginine	Nitric oxide precursor, regulates respiratory chain and oxidative stress in the mitochondria	MELAS	IV arginine improves symptoms during acute MELAS attacks. arginine supplementation increases endothelial function, preventing further stroke-like episodes.	[51]
		Sickle cell disease, vaso-occlusive pain	Phase 2 clinical trial increases mitochondrial activity and reduces oxidative stress.	NCT02536170
L-citrulline	Precursor of arginine	MELAS	Increases the production of NO, as well as concentrations and fluxes of arginine and citrulline.	[161]
deoxythymidine and deoxycytidine substrates	Supplies the nucleoside salvage pathway	TK2d	Improve muscle weakness and ambulation. Discontinuing gastrostomy and mechanical ventilation.	[78]
elamipretide	improves coupling of electron transport chain by Targeting cardiolipin, a phospholipid in the inner mitochondrial membrane	Heart failure	Improvement of cardiac mitochondrial function, and increased efficiency of complex 1 and 4.	[162]
		Barth syndrome cardiomyopathy	Improves mitochondrial bioenergetics and morphology rapidly in induced pluripotent stem cells, normalizes mitochondrial ultrastructure and dynamics	[163]
			Increased left ventricular mass and stroke volume	[164]
		Primary mitochondrial myopathy	MMPOWER-3 clinical trial Primary endpoints were not statistically significant, Class I evidence that elamipretide does not improve the 6-min walk test or fatigue at 24 weeks compared with placebo.	NCT03323749
		LHON	Phase 2 clinical trial Did not achieve primary BCVA outcomes. Improvements in color discrimination, contrast sensitivity and central visual field.	NCT02693119
		Age-related macular degeneration with non-central geographic atrophy	Phase 2 clinical trial Did not reach primary outcomes. Ameliorated mitochondrial-rich ellipsoid zone progressive decline. Improvement of >2 lines visual improvement in low luminance visual acuity.	NCT03891875
Sonlicromanol (KH176)	Antioxidant and redox modulator	Mitochondrial m.3243A>G Spectrum Disorders, e.g., MELAS, MIDD.	KHENERGY Study Phase 2a clinical trial Positive effect on mood and alertness with no other significant parameters.	NCT02909400
		Mammalian model of Leigh syndrome	Improved abnormal gait, motor coordination, learning, and decreased the loss of retinal ganglion cells.	[165]
KL1333	Increase in intracellular NAD	MELAS fibroblasts	Increased ATP decreases ROS and lactate. Improved mitochondrial biogenesis and function.	[166]
		PMD	Phase 1 completed (no posted results)	NCT03888716
			Phase 2 active (no posted results)	NCT05650229
edaravone	antioxidant	MELAS	Scavenges ROS, and inhibits inflammation in cerebrovascular disease, improving vascular function.	[42]
		hyperosmolarity-induced oxidative stress and apoptosis in primary human corneal epithelial cells	Partially attenuated low ATP production induced by hyperosmolarity.	[167]
Bocidelpar (ASP0367)	Modulation of peroxisome proliferator-activated receptor delta, a modulator of cellular energy consumption	PMM	MOUNTAINSIDE study Phase2/3 clinical trial (no results posted)	NCT04641962
Genetic therapy				
GS010	recombinant adeno-associated viral vector serotype 2 containing human wildtype mitochondrial NADH dehydrogenase 4 (ND4) gene	LHON	RESCUE Trial and REVERSE trial Significant improvement of visual acuity in both eyes despite injecting one eye with treatment and the other with a sham injection.	NCT02652767 NCT02652780
			REFLECT Trial Subjects treated bilaterally had better average visual acuity than those treated unilaterally.	NCT03293524
Mitochondrial Augmentation Therapy	Replacing dysfunctional mitochondria with healthy donor mitochondria	Pearson syndrome	Phase1/2 clinical trial	NCT03384420
			Improved mitochondrial function, heteroplasmy, and respiratory capacity. Improved quality of life, aerobic ability, and fine motor functions.	[168]
Mesoangioblasts (MABs)	Lowering the percentage of mtDNA mutational load	m.3243A>G Mutation Carriers	Phase 2 clinical trial Active, recruiting.	NCT05962333
		mtDNA mutated myotubes, m.3271T>C and m.3291T>C mutation	Proportional reduction in mtDNA mutational load in vitro after fusion of wild type MABs.	[169]
		mtDNA point mutation or large-scale deletions	Half the mtDNA carriers have nearly mutation free MABs.	[170]
Allogeneic hematopoietic stem cell transplant	Restoring thymidine phosphorylase enzyme function	MNGIE	Short- and long-term outcomes are influenced by a diagnosis earlier than irreversible gastrointestinal symptoms, a fit matched HLA-donor, and a busulfan-based conditioning regimen.	[171]
			Unfavorable overall outcome pertaining to mortality. Significant improvement in progression and clinical manifestations over time.	[172]

Abbreviations: CPEO: chronic progressive external ophthalmoplegia, 5MTHF: 5-Methyltetrahydrofolate, CSF: cerebrospinal fluid, KSS: Kearns–Sayre syndrome, mtDNA: mitochondrial Deoxyribonucleic acid, LHON: Leber’s hereditary optic neuropathy, MELAS: mitochondrial encephalomyopathy, lactic acidosis, and stroke-like episodes, MRS: magnetic resonance spectroscopy, NAD: nicotinamide adenine dinucleotide, ANT1: adenine nucleotide translocator-1 gene (SLC25A4), TK2d: thymidine kinase 2 deficiency, BCVA: best corrected visual acuity, MIDD: maternally inherited diabetes and deafness, ATP: adenosine triphosphate, ROS: reactive oxygen species, PMD: primary mitochondria disorder, PMM: primary mitochondrial myopathies, NADH: nicotinamide adenine dinucleotide + hydrogen, MNGIE: mitochondrial neurogastrointestinal encephalopathy, HLA: human leukocyte antigens.
